# How Canadian Oncologists Use Oncotype DX for Treatment of Breast Cancer Patients

**DOI:** 10.3390/curroncol28010077

**Published:** 2021-02-04

**Authors:** Xiaofu Zhu, Susan Dent, Lise Paquet, Tinghua Zhang, Daniel Tesolin, Nadine Graham, Olexiy Aseyev, Xinni Song

**Affiliations:** 1 The Ottawa Hospital Cancer Center, University of Ottawa, Ottawa, ON K1H 8L6, Canada; xiaofu@ualberta.ca (X.Z.); susan.dent@duke.edu (S.D.); ngagnon@royalcollege.ca (N.G.); xsong@toh.ca (X.S.); 2 Duke Cancer Institute, Duke University, Durham, NC 27710, USA; 3 Department of Psychology, Carleton University, Ottawa, ON K1S 5B6, Canada; lise.paquet@carleton.ca; 4 Ottawa Hospital Research Institute, The Ottawa Hospital, Ottawa, ON K1Y 4E9, Canada; tizhang@ohri.ca; 5 Northern Ontario School of Medicine, Lakehead University, Thunder Bay, ON P3E 2C6, Canada; dtesolin@nosm.ca; 6 Regional Cancer Care Northwest, Thunder Bay Regional Health Sciences Centre, Thunder Bay, ON P7B 6V4, Canada

**Keywords:** breast cancer, Oncotype DX, clinicopathologic factors

## Abstract

Background: The literature suggests that medical oncologists differ on how they use the Oncotype DX (ODX) genomic assay for making decisions about systemic therapy in breast cancer patients. Given the emergence of data supporting the use of genomic profiling for the prognosis and predicting benefit of chemotherapy, we surveyed medical oncologists in Canada to assess their usage and perception of the ODX assay. Methods: A 34-item survey was distributed to Canadian medical oncologists via the Canadian Association of Medical Oncologists. Data was collected on physician demographics, ODX usage patterns, and physicians’ perception of the impact clinical and pathologic characteristics make on ODX utilization. Results: Response rate was 20.6% with 47 responses received from 228 survey sent. Forty-five responses were eligible for analysis. Sixty-two percent (28/45) of respondents treated predominantly breast cancer, and 60% (27/45) have been in practice for at least 10 years. The most cited reason for using ODX was to avoid giving patients unnecessary chemotherapy (64%; 29/45). Sixty-seven percent (30/45) deferred making treatment decisions until ODX testing was completed. Factors most strongly impacting ODX utilization included: patient request, medical comorbidities and tumor grade. In clinical scenarios, ODX was more frequently selected for patients aged 40–65 (vs. <40 or >65), grade 2 tumors (vs. grade 1 or 3), and Ki-67 index of 10–20% (vs. <10% or >20%). Conclusions: This survey demonstrated that Canadian medical oncologists are preferentially using ODX to avoid giving patients unnecessary chemotherapy. The utilization of ODX is mainly in patients with intermediate clinical and pathologic features.

## 1. Background

Early stage hormone receptor (HR) positive, HER2/neu negative breast cancer is often cured with surgery alone [1]. After surgery, however, there is always a risk of disease recurrence [2]. Physicians must decide whether the risk of recurrence is high, and the patient would benefit from adjuvant chemotherapy or whether the risk of recurrence is low and we can spare the patient the toxicities of overtreatment [1]. Classically, confidence in treatment decision making and patient risk stratification has been based on a group of clinicopathologic factors for determining tumor prognosis such as: age, tumor size, tumor grade, lymph node (LN) involvement, and distant invasion [3].

Studies have shown that adjuvant chemotherapy does improve recurrence-free survival rates regardless of clinicopathologic status, but the absolute benefit of chemotherapy is difficult to determine based on clinical and pathology data alone [1,4]. While tumor size and anatomical spread can be used as markers of disease aggression, these factors are indirect and sometimes do not correlate with actual tumor biological activity [5,6]. Tumor markers such as HR status and HER2/neu overexpression are used by clinicians to get a sense of tumor biology, and thus a prediction of response to treatment [7]. The desire for more direct knowledge about tumor biology led to the development of gene expression profiling technologies to more accurately assess tumor gene activity in order to predict recurrence risk, patterns of metastasis, and treatment response [8]. This desire for better predictive technology is especially strong for younger premenopausal patients who seem to be inflicted with more aggressive disease more of the time [9]. The gene expression profiling product most widely used for breast cancer is the Oncotype DX (ODX) genomic assay (Genomic Health, Inc, Exact Sciences, Redwood City, CA, USA).

ODX assesses 16 cancer related genes and 5 reference genes to develop a score out of 100 called the recurrence score (RS) [10,11]. The National Surgical Adjuvant Breast and Bowel Project (NSABP) B-14 Trial showed that the ODX RS is superior in predicting risk of recurrence compared to classical clinicopathologic markers [4,12]. The NSABP B-20 cohort of HR positive, node negative patients on tamoxifen showed that ODX RS could also accurately predict whom would benefit from adjuvant chemotherapy and who would not [4,13]. The recent large prospective TailorX trial has confirmed that chemotherapy should be spared for HR positive, HER2 negative early stage breast cancer patients over 50 years old and a RS ≤ 25 or those 50 years or less and a RS ≤ 15 [14].

Most recently, ODX RS has shown promising results for use in node positive disease (1–3 nodes) as well. A retrospective analysis of the randomized phase 3 SWOG-8814 trial showed the prognostic power of a high ODX RS for detecting high risk tamoxifen patients with positive nodes [15,16]. The ATAC trial and prospective PlanB trial show support of using RS in node positive disease but these trials still suffer from either short follow-up time or small sample sizes [17,18,19]. Clinicians are urgently awaiting concrete clinical recommendations from the large ongoing RxPONDER trial which enrolls patients with ER-positive, HER2-negative disease with 1–3 positive LNs [16,20,21,22].

There have even been recent updates to guidelines, due to the results of the TailorX and MINDACT Trials and early results from the RxPONDER Trial, for treatment decisions in patients with intermediate RS scores and LN positive disease [23,24].

Despite the international recommendations to incorporate ODX into clinical decision making, the literature suggests that the utilization of ODX varies between different practitioners and populations. A single center study in Ottawa, Canada showed that for HR positive, HER2 negative early stage breast cancer patients, oncologists mainly used age (50–64 years), tumor size (10.1–20 mm), and grade (2) for deciding when to order ODX [25]. Another American study conducted in New York showed similar results [26]. A German study showed significant differences between oncologist decision making came from a subgroup of patients with Ki-67 > 14%, tumor sizes larger than pT2, pN1, and postmenopausal status [27]. An Australian study of 151 patients showed oncologists changed adjuvant chemotherapy recommendations for 24–26% of the patients after reviewing ODX results. These oncologists were two times more likely to remove adjuvant chemotherapy than adding it in node negative disease and four times more likely to remove chemotherapy in node positive disease [28]. Similar results were found in a Spanish study cohort [29]. In Ontario, Canada a prospective study also showed a 36% reduction in chemotherapy in those with 1–3 positive nodes after the ODX assay, displaying its prognostic impact even in those with node positive disease [30].

There is evidence in the literature that suggests not all oncologists utilize the assay uniformly, despite authors reporting direct changes to management in response to ODX RS in 24–44% of patients [20,28,29,30,31,32,33,34,35,36]. Interviews on 14 oncologists in Toronto, Ontario discovered that they valued the test as a treatment-decision tool but there are some concerns about cost, over-reliance, overuse, inappropriate use, and patient’s limited understanding of the test [37]. A review of ten studies from eight different populations showed that recurrence testing gave some women anxiety, some of which was attributed to poor comprehension of the results. Most women in the study, however, would recommend testing to others and were satisfied with the process [38]. This differs in one American study showed that 15% of oncologists would never or rarely order ODX testing at all [39]. Certainly access and utilization will differ in different populations due to various healthcare models, access to resources, and demographics [40].

There are several factors reported that cause discrepancies in how ODX is used. These include oncologist experience, testing of patients under 50 years old, decision making with intermediate RS values (16–25), ordering ODX when clinicopathologic features already suggest low risk, the applicability of results for non-classical chemotherapies, and the cost or availability of the test itself [18,27,40,41,42]. ODX is another tool that can be used to aid clinicians reduce their uncertainty about the recurrence of disease but there is clear variation in how oncologists rely on ODX results. Since ODX is a relatively expensive assay its use by oncologists may also be persuaded by system funding.

Considering the rapidly evolving evidence surrounding ODX, we were particularly interested in how ODX was utilized in Canada before the results of TailorX and RxPONDER. Our public health care system has been adapting to fund the utilization of this test based on emerging data. Here we report the results and evaluation of a 34-item survey that was distributed to Canadian medical oncologists to elucidate their ODX usage patterns and factors influencing their decision making surrounding systemic treatment of early stage breast cancer patients.

## 2. Methods

A 34-item survey was distributed to Canadian medical oncologists via the Canadian Association of Medical Oncologists. A total of 228 surveys were distributed during the time period of December 2012 and August 2013. Data was collected on physician demographics, current ODX usage patterns, and physicians’ perception of the impact of various clinical and pathologic characteristics on their decision to utilize ODX in early stage breast cancer patients. We also examined which factors were most important in decision making surrounding systemic therapy. The survey included case scenarios to understand medical oncologist decision making in realistic clinical settings.

## 3. Results

The survey response rate was 20.6% with 47 responses from 228 surveys sent to Canadian medical oncologists. Respondents responded to about 81% of the questions within the survey on average. About 60% of the respondents treated patients with breast cancer predominately, with 73% of them working out of academic centers. About half of the respondents worked in Ontario (46%). The distribution of the respondents was approximately evenly divided in terms of gender (52% female; 48% male) and years of work experience (40% <10 years; 60% >10 years) (Table 1).

**Figure 1:**
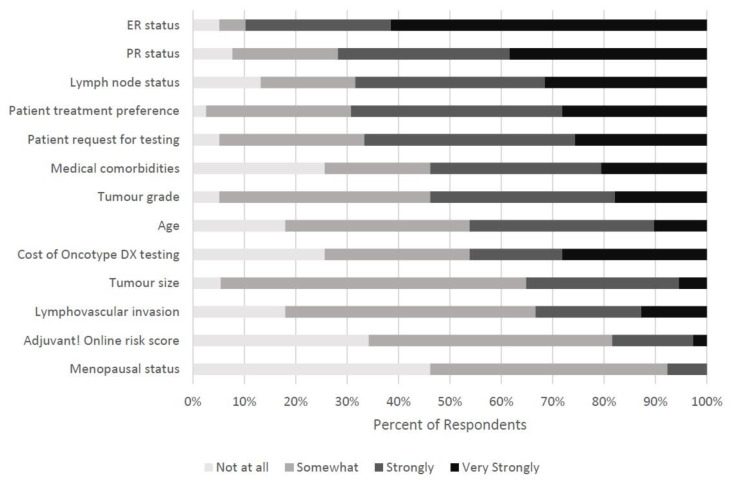
Survey results for questions related to how strongly the respondents felt the following factors influence their use of ODX.

When the medical oncologists were asked how strongly clinicopathologic factors influenced their use of ODX they answered on a descriptive Likert scale ranging from “Not at all” to “Very Strongly”. To analyze the data, the categories of “Not at all” and “Somewhat” represented weak importance while “Strongly” and “Very Strongly” represented strong importance (Table 2). At least 60% of respondents felt in decreasing order that ER status (90%), PR status (72%), patient treatment preference (69%), LN status (68%), and patient request for testing (67%) had strong influence on their ODX use. Very few respondents felt that menopausal status (8%), Adjuvant! Online risk score (18%), lymphovascular invasion (33%), and tumor size (35%) had strong importance. Respondents were split on whether the cost of ODX testing, age or tumor grade had strong influence on their ODX utilization (Figure 1).

We asked respondents more detailed questions about these clinicopathologic categories and how frequently they use ODX in more specific situations (Table 3). We found most respondents would at least “Sometimes” order ODX for ER + ve/PR + ve (40%) and ER + ve/PR−ve (45%) patients. Conversely, most respondents said they would “Rarely” or “Never” order ODX for ER-ve/PR-ve patients (70% total).

Many of our respondents are ordering ODX for their LN negative patients (“Often” or “Always” (40%) and those with microscopic metastasis (“Often” and “Always” (19%). Respondents “Rarely” or “Never” order ODX for patients with 1–3 disease positive nodes (85%) or greater than 3 positive nodes (100%).

Respondents tended to order ODX “Rarely” or “Never” for the age categories “<35”, (73%), “35–40” (50%), and “>70” (74%), for patient with Moderate Health Issues (45%) or Major Health Issues (85%). There was a large amount of disagreement for whether they would order ODX for age categories “41–50”, “51–60”, and “61–70”. Respondents were split on use of menopausal status. Certainly, a large proportion of the respondents would consider menopausal status “Sometimes” (pre, peri, and post-menopausal status; 38%, 45%, and 43%, respectively) but the rest could not agree on its importance.

The respondents tended to order ODX for patients with tumors of moderate grade and size. Most said they would order ODX for Grade 2 tumors “Sometimes” (40%) or “Often”, 34%). Similar results were found for moderate tumor sizes as respondents would “Sometimes” order ODX for sizes 1.1–2 cm (35%) and 2.1–5 cm (48%). They indicated they would “Rarely” or “Never” order it for Grade 1 tumors (65%), Grade 3 tumors (50%), tumors 0–1 cm (75%), and tumors > 5 cm (82.5%).

**Figure 2:**
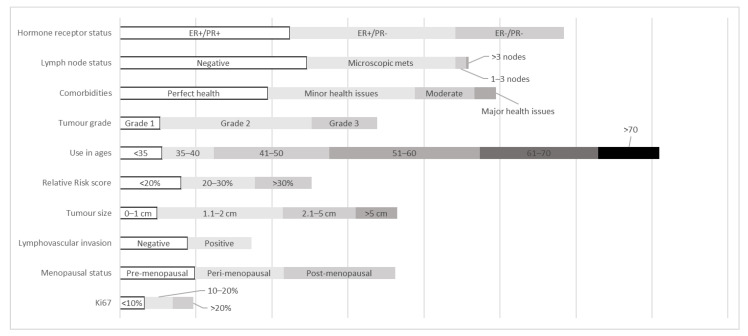
The frequency of ODX utilization for different clinicopathologic factors. The data has been transformed by applying an exponential weighting factor to better visualize which factors have the greatest importance to the respondents.

According to the survey, respondents generally did not consider relative risk score, lymphovascular invasion or Ki-67 status as important factors for ordering ODX. (Figure 2).

The medical oncologist respondents also had case evaluations to assess (Table 4). Case 1 has patients of varying age categories to treat with all other clinicopathologic factors constant. Most (84%) would treat patients less than 40 years old with chemotherapy and endocrine therapy but tended towards endocrine therapy alone for the patients of increasing age (51–65, 30%; 66–75, 70%; and >75, 97%). A similar trend was seen for ordering ODX as they demonstrated higher ordering rates for younger patients (<40, 68%; 40–50, 87%; 51–65, 76%; 66–75, 57%; and >75, 8%).

In Case 2, opposite trends were revealed for patients with increasing tumor grade. As tumor grade increased respondents tended to transition from endocrine only therapy to endocrine therapy plus chemotherapy (Grade 1, 86%; Grade 2, 24%; Grade 3, 3% endocrine only therapy). Respondents were more than twice as likely to be Unsure for Grade 2 tumors versus Grade 1 or 3 (19% unsure of treatment versus 8% and 8%, respectively). About half of the respondents would order ODX for tumor grades 1 and 3 (54% and 62%, respectively) but almost all would for tumor Grade 2 (89%).

In Case 3, respondents indicated that they prefer to treat most patients suffering from significant comorbidities with endocrine therapy alone (84%) and are unlikely to order ODX (19%). For patients with lobular carcinoma, treatment was dictated by tumor grade, with endocrine therapy being the treatment of choice for Grade 1 patients (92%), whereas more uncertainty being present for Grade 2 patients (chemotherapy and endocrine therapy, 51%; endocrine therapy alone, 27%; and unsure, 19%). Almost all respondents agreed that grade 3 lobular carcinoma patients should be treated with chemotherapy plus endocrine therapy (75%). Respondents were split on whether they would order ODX for Grade 2 (60%) or Grade 3 (65%) but few would order it for Grade 1 lobular carcinoma patients (32%).

Respondents would order mainly chemotherapy and endocrine therapy for Grade 2 tumors with positive lymphovascular invasion (81%) or PR-ve status (70%). They also tended to order ODX for these two cases (78%, 92%, respectively).

In Case 4, many respondents were unsure how they would treat patients of varying Ki-67 status. For Ki-67 < 10%, respondents would mostly treat with endocrine therapy alone (54%) but many were unsure (22%). For Ki-67 status of 10–25% and >25%, most would treat with chemotherapy and endocrine therapy (38%, 70%, respectively) but many were still unsure (41%, 16%, respectively). Respondents were split on whether they would order ODX for these patients.

**Figure 3:**
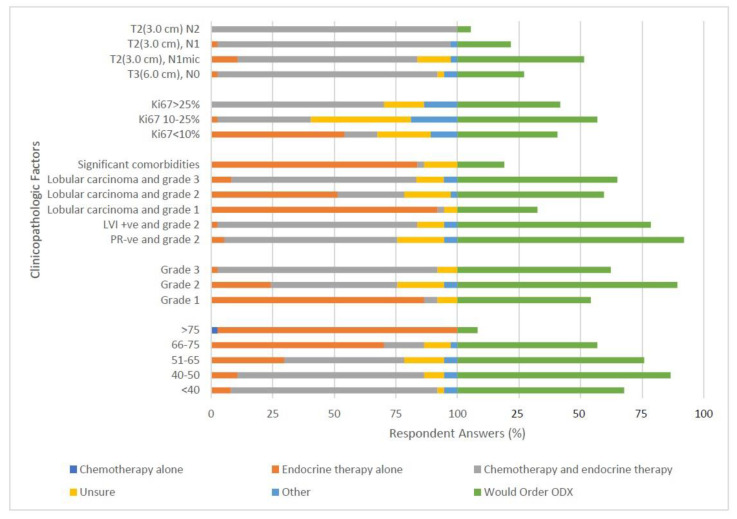
Treatment decisions made by the respondents for different theoretical patient cases. Included is data related to whether the respondent would order ODX for the theoretical patient cases as well.

Finally, in Case 5, respondents indicated they would treat with chemotherapy and endocrine therapy for patients of T2 (3 cm) tumor size with increasing nodal status (T2, N1mic, 73%; T2, N1, 95%; T2, N2, 100%) or a patient with negative nodal spread but a T3 (6 cm) tumor size (89%). Half the respondents would order ODX for the patient with T2, N1mic (51%) but few would order ODX for the more extensive nodal status tumors (Figure 3).

## 4. Discussion

For the medical oncologists who responded to this survey to be eligible for this study they were required to treat breast cancer patients in their practice. Although there were only 47 responses to the survey this is still quite representative of the medical oncology population in Canada. According to a recent report done by the Canadian Medical Association there are 625 medical oncologists practicing in Canada [43]. According to the demographic results over 70% of the respondents had practices with at least half of the patients being treated for breast cancer. This is not surprising given the high prevalence of breast cancer and the fact that over 70% of the respondents worked in academic centers where oncologists tend to focus on specific sites. The distribution of respondents in terms of gender and years in practice is fairly even but there is a disproportionate number of physicians from Ontario (47%) compared to elsewhere in Canada. This is important because funding of ODX is different in each province but is largely funded in Ontario. Respondents have indicated that only 53% are certain their province funds ODX while 34% do not think their province does and 13% are not sure. Cost can be a barrier when ordering ODX, especially when its results are deemed to not affect treatment decisions.

At the time this survey was distributed respondents felt ODX was a tool best used to avoid giving patients unnecessary chemotherapy or to determine the absolute benefit of chemotherapy. This is not surprising as 43% of respondents indicated they defer treatment decisions “Often” to “Always”. Up to 45% consider ODX results as information that influences treatment decision more than other factors. The respondents consider ODX less important for providing prognosis or for convincing patients they may benefit from chemotherapy.

It is obvious from the results that there are clinicopathologic factors that strongly influence the respondent’s use of ODX and some that have less importance. The factors that were considered the strongest were HR status, LN status, patient treatment preference, and patient request for testing. Respondents could not agree on the importance of medical comorbidities, tumor grade, age, and cost of ODX. Most considered tumor size, lymphovascular invasion, the Adjuvant! Online risk score, and menopausal status to have little importance. Like ODX, HR status gives information about tumor biology, while LN status remains an indirect measure of the aggressiveness of a tumor. At the time of this survey it appears medical oncologists rely heavily on LN status for making treatment decisions despite more recent information suggesting some LN positive disease does not require aggressive chemotherapy.

Although HR status and LN status appear to be the clinicopathologic factors that most strongly influence ODX use, it is important to note for which specific patients ODX will be ordered. ODX is utilized most in the HR positive patient population which is consistent with current evidence for the utilization of ODX. Most physicians use ODX in node negative or microscopic metastasis. This is most likely influenced by public funding criteria and evidence available to physicians at the time of this survey.

The data suggests oncologists use ODX mostly to aid with treatment decisions in patients that have less than moderate health comorbidities because these patients can handle the therapy and in patients with intermediate type risk factors. They would order ODX most for grade 2 tumors, patients 41–70 years old and tumor size 1.1–2 cm. ODX was somewhat important for lymphovascular invasion and menopausal status but the status of these categories did not change the respondent’s decision regarding ODX frequency of use. Ki-67 status appeared to be universally unimportant to the respondents. This is maybe due to the lack of standard testing and resources for routine Ki67 reporting by pathology laboratories.

The case examples given to the medical oncologist respondents gave us insight into how different clinicopathologic features affect treatment decision in early stage breast cancer patients. When given a patient controlled for all factors except age the respondents tended to favor treating with endocrine therapy alone for extremes of age compared to a more aggressive regimen of chemotherapy combined with endocrine therapy for younger patients that could tolerate it better. As seen before they only ordered ODX for the patients that they believed could tolerate aggressive treatment and still had a long-life expectancy. This is seen again in subsequent cases with grade 2 tumors; the respondents have higher amounts of uncertainty and variation about treatment decision and larger instances of ordering ODX to help with treatment decision. Contrast this with situations where the risk is obviously high or low and treatment decisions become more agreed upon.

Interestingly, in every instance of node positive disease or in T3 (6.0 cm), N0 disease the respondent’s use of ODX was universally low and treatment decision was almost unanimously chemotherapy and endocrine therapy combined. This again shows how much weight the oncologists put on LN status as a surrogate for aggressive disease. It is important to note, however, that this survey was distributed before the TAILORX trial and RxPONDER trial which suggest ODX has the ability to differentiate tumors that would benefit from chemotherapy and those that would not, even in node positive disease [16]. It would be prudent to re-distribute this survey to assess the changes in practice that may be associated with updated trial data.

This study has some obvious limitations. The demographics show the medical oncologists were disproportionately from Ontario, a province known to fund ODX whereas the others have more limitations on funding. The results would have more strongly represented Canada if there was a larger number of oncologists responding from other provinces, although, Ontario employs about 38% of Canada’s oncologists [43]. The total sample size of 47 respondents does limit the statistical power of our results, however, as mentioned above we believe this sample is quite representative of the small population of medical oncologists in Canada using ODX for breast cancer.

## 5. Conclusions

This is a survey given out to Canadian medical oncologists to assess their use of ODX in early stage cancer patients. The respondents were well varied in gender, work experience, proportion of breast cancer patients treated and access to provincially funded ODX. They were disproportionately from Ontario and mostly from academic cancer centers which could have influenced results. They feel ODX is a tool best used to avoid giving patients unnecessary chemotherapy or to determine the absolute benefit of chemotherapy.

The oncologists surveyed felt that the clinicopathologic factors that influenced their decision the most for ordering the ODX test was in patients with positive HR status, negative LN status, perfect to minor health issues, grade 2 tumors that are 1.1–2 cm in size in patients that are 51–60 years of age, especially those that are post-menopausal. Relative Risk score, lymphovascular invasion, and Ki67 status did not have much influence on their decisions.

When it came to making treatment decisions, the respondents tended to treat older patients and those with less aggressive tumor qualities with endocrine therapy alone while treating younger, healthy patients with more aggressive tumor qualities with endocrine therapy plus chemotherapy. They tended to order ODX more for intermediate risk factors such as grade 2 tumors or patients that are between 50 and 65 years of age.

For large tumors or tumors with any nodal spread the respondents almost always treated with both chemotherapy and endocrine therapy while rarely ordering ODX. This would be consistent with the available data at the time of the survey and public funding criteria for ODX in LN negative, HR positive cancer only. Given new data from the prospective clinical trials for ODX utilization in LN positive disease a future survey can help assess the impact new trial data will have on the treatment decisions made by medical oncologists in Canada.

## Figures and Tables

**Table 1:** Survey respondent demographic data.

Oncologist Demographic and Practice Type	Survey Answer Frequency (%)
Percentage of breast cancer patients	
0–25%	10 (21.3)
25–50%	9 (19.2)
50–75%	11 (23.4)
>75%	17 (36.2)
Province you practice	
Alberta	4 (8.5)
British Columbia	10 (21.3)
Manitoba	2 (4.3)
New Brunswick	1 (2.1)
Newfoundland and Labrador	4 (8.5)
Nova Scotia	1 (2.1)
Ontario	22 (46.8)
Prince Edward Island	1 (2.1)
Quebec	2 (4.3)
Practice Setup	
Academic Cancer Center	33 (73.3)
Community Cancer Center	12 (26.7)
Years in Practice	
0–10 years	19 (40.4)
10–20 years	11 (23.4)
>20 years	17 (36.2)
Gender	
Female	24 (52.2)
Male	22 (47.8)
Province fund the use of Oncotype Dx	
Yes	25 (53.2)
No	16 (34.0)
Limited/not sure	6 (12.8)

**Table 2:** Survey question: How strongly do the following factors influence your use of Oncotype DX? (Not at all, Somewhat, Strongly or Very Strongly).

Characteristic—No (%)	Not at All	Somewhat	Strongly	Very Strongly
ER status	2 (5.1)	2 (5.1)	11 (28.2)	24 (61.5)
PR status	3 (7.7)	8 (20.5)	13 (33.3)	15 (38.5)
Lymph node status	5 (13.2)	7 (18.4)	14 (36.8)	12 (31.6)
Patient treatment preference	1 (2.6)	11 (28.2)	16 (41.0)	11 (28.2)
Patient request for testing	2 (5.1)	11 (28.2)	16 (41.0)	10 (25.6)
Medical comorbidities	10 (25.6)	8 (20.5)	13 (33.3)	8 (20.5)
Tumor grade	2 (5.1)	16 (41.0)	14 (35.9)	7 (17.9)
Age	7 (17.9)	14 (35.9)	14 (35.9)	4 (10.3)
Cost of Oncotype DX testing	10 (25.6)	11 (28.2)	7 (17.9)	11 (28.2)
Tumor size	2 (5.4)	22 (59.5)	11 (29.7)	2 (5.4)
Lymphovascular invasion	7 (17.9)	19 (48.7)	8 (20.5)	5 (12.8)
Adjuvant! Online risk score	13 (34.2)	18 (47.4)	6 (15.8)	1 (2.6)
Menopausal status	18 (46.2)	18 (46.2)	3 (7.7)	0 (0.0)

**Table 3:** Survey question: How frequently do you use Oncotype DX for the following clinical and pathologic features? (Never, Rarely, Sometimes, Often, Always).

Characteristic—No (%)	Never	Rarely	Sometimes	Often	Always
Hormone Stat					
ER+/PR+	2 (5.0)	9 (22.5)	16 (40.0)	10 (25.0)	3 (7.5)
ER+/PR−	2 (5.0)	8 (20.0)	18 (45.0)	9 (22.5)	3 (7.5)
ER−/PR−	14 (35.0)	14 (35.0)	3 (7.5)	7 (17.5)	2 (5.0)
Lymph node statu					
Negative	1 (2.5)	7 (17.5)	16 (40.0)	13 (32.5)	3 (7.5)
Microscopic metastasis	4 (10.0)	9 (22.5)	18 (45.0)	6 (15.0)	3 (7.5)
1–3 nodes	24 (60.0)	10 (25.0)	6 (15.0)	0 (0.0)	0 (0.0)
>3 nodes	35 (87.5)	5 (12.5)	0 (0.0)	0 (0.0)	0 (0.0)
Medical comorbidities					
Perfect Health	2 (5.0)	7 (17.5)	18 (45.0)	11 (27.5)	2 (5.0)
Minor Health Issues	2 (5.0)	8 (20.0)	17 (42.5)	11 (27.5)	2 (5.0)
Moderate Health Issues	3 (7.9)	14 (36.8)	15 (39.5)	6 (15.8)	0 (0.0)
Major Health Issues	16 (40.0)	18 (45.0)	4 (10.0)	2 (5.0)	0 (0.0)
Tumor grade					
Grade 1	9 (22.5)	17 (42.5)	10 (25.0)	4 (10.0)	0 (0.0)
Grade 2	2 (5.0)	8 (20.0)	16 (40.0)	12 (30.0)	2 (5.0)
Grade 3	6 (15.0)	14 (35.0)	12 (30.0)	8 (20.0)	0 (0.0)
Age					
<35	8 (20.0)	21 (52.5)	6 (15.0)	5 (12.5)	0 (0.0)
35–40	3 (7.5)	17 (42.5)	15 (37.5)	5 (12.5)	0 (0.0)
41–50	2 (5.0)	11 (27.5)	15 (37.5)	11 (27.5)	1 (2.5)
51–60	2 (5.0)	9 (22.5)	15 (37.5)	12 (30.0)	2 (5.0)
61–70	2 (5.1)	10 (25.6)	15 (38.5)	11 (28.2)	1 (2.6)
>70	10 (25.6)	19 (48.7)	6 (15.4)	3 (7.7)	1 (2.6)
Relative Risk Score					
10 year RR < 20%	4 (10.0)	16 (40.0)	13 (32.5)	7 (17.5)	0 (0.0)
10 year RR 20–30%	6 (15.0)	10 (25.0)	15 (37.5)	9 (22.5)	0 (0.0)
10 year RR > 30%	12 (30.0)	11 (27.5)	10 (25.0)	7 (17.5)	0 (0.0)
Tumor size					
0–1 cm	5 (12.5)	25 (62.5)	6 (15.0)	4 (10.0)	0 (0.0)
1.1–2 cm	2 (5.0)	10 (25.0)	14 (35.0)	13 (32.5)	1 (2.5)
2.1–5 cm	3 (7.5)	10 (25.0)	19 (47.5)	8 (20.0)	0 (0.0)
>5 cm	15 (37.5)	18 (45.0)	6 (15.0)	0 (0.0)	1 (2.5)
Lymphovascular invasion					
Negative	4 (10.5)	10 (26.3)	17 (44.7)	7 (18.4)	0 (0.0)
Positive	6 (15.4)	11 (28.2)	15 (38.5)	7 (17.9)	0 (0.0)
Menopausal Status					
Premenopausal	1 (2.5)	15 (37.5)	15 (37.5)	9 (22.5)	0 (0.0)
Perimenopausal	2 (5.0)	9 (22.5)	18 (45.0)	11 (27.5)	0 (0.0)
Postmenopausal	2 (5.0)	10 (25.0)	17 (42.5)	10 (25.0)	1 (2.5)
Ki67					
<10%	14 (42.4)	8 (24.2)	10 (30.3)	1 (3.0)	0 (0.0)
10–20%	13 (39.4)	6 (18.2)	13 (39.4)	1 (3.0)	0 (0.0)
>20%	18 (54.4)	7 (21.2)	7 (21.2)	1 (3.0)	0 (0.0)

**Table 4:** Respondent treatment decisions based on several different theoretical case scenarios. The respondents were also asked whether or not they would order ODX in the various scenarios.

Characteristic—No (%)	Chemo Alone	Endocrine Tx Alone	Chemo + Endocrine	Unsure	Other	Would Use ODX
Case 1: Age–no (%)						
<40	0 (0.0)	3 (8.1)	31 (83.8)	1 (2.7)	2 (5.4)	25 (67.6)
40–50	0 (0.0)	4 (10.8)	28 (75.7)	3 (8.1)	2 (5.4)	32 (86.5)
51–65	0 (0.0)	11 (29.7)	18 (48.6)	6 (16.2)	2 (5.4)	28 (75.7)
66–75	0 (0.0)	26 (70.3)	6 (16.2)	4 (10.8)	1 (2.7)	21 (56.8)
>75	1 (2.7)	36 (97.3)	0 (0.0)	0 (0.0)	0 (0.0)	3 (8.1)
Case 2: Tumor Grade–no (%)						
Grade 1	0 (0.0)	32 (86.5)	2 (5.4)	3 (8.1)	0 (0.0)	20 (54.1)
Grade 2	0 (0.0)	9 (24.3)	19 (51.4)	7 (18.9)	2 (5.4)	33 (89.2)
Grade 3	0 (0.0)	1 (2.7)	33 (89.2)	3 (8.1)	0 (0.0)	23 (62.2)
Case 3: Other Risk Factors–no (%)						
PR-ve and grade 2	0 (0.0)	2 (5.4)	26 (70.3)	7 (18.9)	2 (5.4)	34 (91.9)
LVI +ve and grade 2	0 (0.0)	1 (2.7)	30 (81.1)	4 (10.8)	2 (5.4)	29 (78.4)
Lobular carcinoma and grade 1	0 (0.0)	34 (91.9)	1 (2.7)	2 (5.4)	0 (0.0)	12 (32.4)
Lobular carcinoma and grade 2	0 (0.0)	19 (51.4)	10 (27.0)	7 (18.9)	1 (2.7)	22 (59.5)
Lobular carcinoma and grade 3	0 (0.0)	3 (8.3)	27 (75.0)	4 (11.1)	2 (5.6)	24 (64.9)
Significant comorbidities	0 (0.0)	31 (83.8)	1 (2.7)	5 (13.5)	0 (0.0)	7 (18.9)
Case 4: Ki67 Status–no (%)						
Ki67 < 10%	0 (0.0)	20 (54.1)	5 (13.5)	8 (21.6)	4 (10.8)	15 (40.5)
Ki67 10–25%	0 (0.0)	1 (2.7)	14 (37.8)	15 (40.5)	7 (18.9)	21 (56.8)
Ki67 > 25%	0 (0.0)	0 (0.0)	26 (70.3)	6 (16.2)	5 (13.5)	15 (41.7)
Case 5: Tumor Size and Nodal Status–no (%)						
T3(6.0 cm), N0	0 (0.0)	1 (2.7)	33 (89.2)	1 (2.7)	2 (5.4)	10 (27.0)
T2(3.0 cm), N1mic	0 (0.0)	4 (10.8)	27 (73.0)	5 (13.5)	1 (2.7)	19 (51.4)
T2(3.0 cm), N1	0 (0.0)	1 (2.7)	35 (94.6)	0 (0.0)	1 (2.7)	8 (21.6)
T2(3.0 cm) N2	0 (0.0)	0 (0.0)	37 (100.0)	0 (0.0)	0 (0.0)	2 (5.4)

## Abbreviations

ER	estrogen receptor
HER2	human epidermal growth factor receptor 2
HR	hormone receptor
LN	lymph node
ODX	Oncotype DX
PR	progesterone receptor
RS	recurrence score
